# Tenofovir alafenamide for prevention and treatment of hepatitis B virus reactivation and de novo hepatitis

**DOI:** 10.1002/jgh3.12636

**Published:** 2021-08-19

**Authors:** Kento Inada, Shun Kaneko, Masayuki Kurosaki, Koji Yamashita, Sakura Kirino, Leona Osawa, Yuka Hayakawa, Shuhei Sekiguchi, Mayu Higuchi, Kenta Takaura, Chiaki Maeyashiki, Nobuharu Tamaki, Yutaka Yasui, Jun Itakura, Yuka Takahashi, Kaoru Tsuchiya, Hiroyuki Nakanishi, Ryuichi Okamoto, Namiki Izumi

**Affiliations:** ^1^ Department of Gastroenterology and Hepatology Musashino Red Cross Hospital Tokyo Japan; ^2^ Department of Gastroenterology and Hepatology Tokyo Medical and Dental University Tokyo Japan

**Keywords:** de novo hepatitis, hepatitis B virus reactivation, tenofovir alafenamide

## Abstract

**Background and Aim:**

Administration of tenofovir alafenamide (TAF) as prevention or treatment of hepatitis B virus (HBV) reactivation is not well known. The aim of this study is to reveal the efficacy and safety of TAF against HBV reactivation.

**Methods:**

Entecavir (ETV) and TAF were given to 66 and 11 patients, respectively, as prophylaxis against or treatment of HBV reactivation during chemotherapy or immune suppression therapy from January 2010 to June 2020. The antiviral effects and safety were assessed.

**Results:**

At week 24, the antiviral effects on patients receiving ETV and TAF were similar in terms of reduction of HBV DNA (−2.83 ± 1.45log IU/mL *vs* −3.05 ± 2.47log IU/mL; *P* = 0.857) and achieving undetectable levels of HBV DNA (78.8 *vs* 90.9%; *P* = 0.681). There was no significant difference in the decrease in the estimated glomerular filtration rate (eGFR) between the two groups (−0.62 ± 11.2 mL/min/1.73 m^2^ *vs* −3.67 ± 13.2 mL/min/1.73 m^2^; *P* = 0.291).

**Conclusion:**

TAF is safe and effective against HBV reactivation.

## Graphical abstract

The aim of this study is to reveal the efficacy and safety of tenofovir alafenamide (TAF) as prophylaxis against or treatment of hepatitis B virus (HBV) reactivation. The antiviral effects in terms of HBV DNA reduction on patients receiving entecavir and TAF were similar, and there was no significant difference in renal dysfunction between both groups. TAF is safe and effective against HBV reactivation.

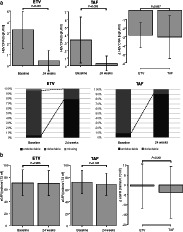

## Introduction

Hepatitis B virus (HBV) reactivation is the reactivation of HBV DNA during cytotoxic or immunosuppressive therapy in patients who were previously treated for an HBV infection.^1^ HBV reactivation can be asymptomatic but oftentimes it is followed by a clinical flare characterized by a substantial increase of serum transaminase levels and histologic evidence of active inflammation. Occasionally, such flares may lead to fatal hepatic failure.^2^, ^3^, ^4^ Once HBV infects the host's cells, the covalently closed circular (ccc) DNA remains stable in the infected cells and serves as a template for viral replication.^5^, ^6^ Therefore, patients with a past history of an HBV infection are at risk for HBV reactivation.^1^, ^7^, ^8^

HBV surface antigen (HBsAg)‐positive patients are at high risk of HBV reactivation and should receive nucleoside and nucleotide analog (NA) therapy before the initiation of immunosuppressive or cytotoxic therapy. HBsAg‐negative and anti‐HBc‐positive patients with a resolved HBV infection have a lower risk of HBV reactivation than HBsAg‐positive patients. Depending on the clinical situation and feasibility of close monitoring, these patients can begin anti‐HBV prophylaxis or monitoring with the intent of initiating an on‐demand antiviral therapy at the first sign of reactivation. These are described in the guidelines of the Japan Society of Hepatology (JSH),^9^ The American Association for the Study of Liver Diseases (AASLD),^10^ and The European Association for the Study of the Liver (EASL).^11^ Recently, atezolizumab and bevacizumab were approved for first‐line chemotherapy for patients with advanced hepatocellular carcinoma (HCC).^12^ The number of patients who have immune checkpoint inhibitors on a background of viral hepatitis is markedly increasing. A study was conducted on HBV reactivation in HBsAg‐positive patients who received anti‐PD‐1/PD‐L1 antibody.^13^ American Society of Clinical Oncology (ASCO) recommends prophylactic antiviral therapy to be initiated in HBsAg‐positive patients prior to chemotherapy, including immune checkpoint inhibitors.^14^ There have been cases of HBV reactivation and severe liver damage after administration of immune checkpoint inhibitors in HBsAg‐positive cases, although further research is required to understand this phenomenon. Therefore, the present study is focused on HBV reactivation.

Recently, tenofovir alafenamide (TAF), which was designed to have greater plasma stability compared with tenofovir disoproxil fumarate (TDF), was approved for clinical application. In previous studies, TAF has proven to be as effective as TDF and has led to the continuous improvement in renal and bone safety in the treatment of patients with chronic hepatitis B.^15^, ^16^, ^17^, ^18^

Several NA studies have shown that prophylaxis with entecavir (ETV) and TDF was significantly associated with a lower risk of HBV reactivation.^19^, ^20^, ^21^, ^22^, ^23^ However, there are no studies evaluating the efficacy of TAF as prophylaxis against or treatment for HBV reactivation. It is necessary that a study be conducted to compare the efficacy and safety between ETV and TAF as prophylaxis against or treatment for HBV reactivation. The aim of this study is to determine the efficacy and safety of TAF as prophylaxis against or treatment for HBV reactivation through case series analysis.

## Methods

From January 2010 to June 2020, 77 patients in Musashino Red Cross Hospital received NA therapy (ETV or TAF) as prophylaxis against or treatment for HBV reactivation. Before the study, baseline levels of aspartate aminotransferase (AST), alanine aminotransferase (ALT), HBeAg, HBsAg, HBV core antibody (HBcAb), HBsAb, serum HBV DNA, creatinine, and estimated glomerular filtration rate (eGFR) were measured. Written informed consent was obtained from all patients. HBV DNA was quantified by real‐time polymerase chain reaction (Roche). HBeAg and HBV genotypes were confirmed by commercially available enzyme immunoassay kits. To analyze the distribution of continuous variables, Student's *t*‐test or the Mann–Whitney *U*‐test was performed. To analyze the changes in continuous variables, the paired *t*‐test or the Wilcoxon signed rank test was used. Fisher's exact test was conducted for analyses of categorical variables. Statistical significance was defined as a *P* value <0.05. All statistical analyses were carried out with EZR (Saitama Medical Center, Jichi Medical University, Saitama, Japan). The bars in all the graphs represent the mean ± SD values.

All procedures carried out in studies involving human participants were in accordance with the ethical standards of the institutional research committee and with the Helsinki Declaration. Our institute waived institutional review board approval for case reports.

## Results

### A representative case

**Figure 1:**
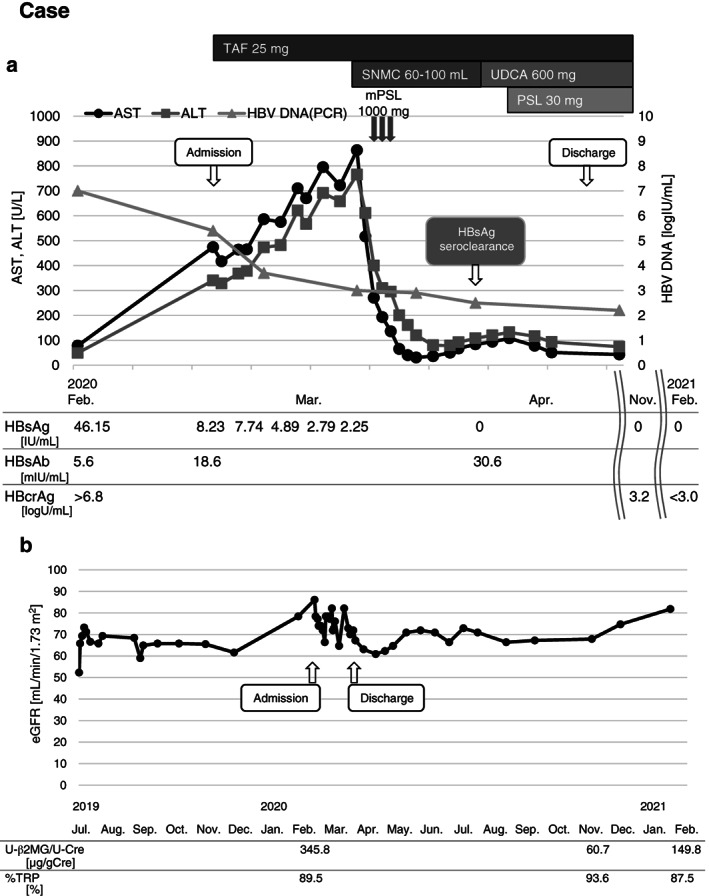
Clinical course of the patient with de novo hepatitis. (a) The changes in serum levels of aspartate aminotransferase (AST), alanine aminotransferase (ALT), and hepatitis B virus (HBV) DNA are shown in the graph along with the levels of hepatitis B surface antigen (HBsAg), hepatitis B core antibody (HBcAb), and hepatitis B core related antigen (HBcrAg). (b) The changes in the estimated glomerular filtration rate (eGFR) of the patients are shown in the graph along with the levels of urinary β2 microglobulin (U‐β2MG) and % tubular reabsorption of phosphate (%TRP). Urinary function did not worsen after the initiation of TAF. mPSL, methyl prednisolone; PSL, prednisolone; SNMC, stronger neo‐minophagen C; TAF, tenofovir alafenamide fumarate; UDCA, ursodeoxycholic acid

A 77‐year‐old male was diagnosed with primary gastric diffuse large B cell lymphoma (DLBCL). He underwent gastric surgery and received adjuvant chemoradiotherapy consisting of rituximab, cyclophosphamide, doxorubicin, vincristine, and prednisolone (R‐CHOP). He also underwent radiation therapy (30Gy in 15 fractions). He was negative for HBsAg and positive for HBcAb with undetectable HBV DNA at baseline. After 2 months of chemotherapy, his serum HBV DNA and HBsAg increased to 7.0 Log IU/mL and 46.15 IU/mL. Furthermore, his serum AST and ALT levels increased to 474 U/L and 340 U/L, respectively. He was diagnosed with de novo hepatitis B (Fig. 1a) and was started on TAF 25 mg/day. The level of liver enzymes was continuously elevated, and on day 21, the ammonia level was also elevated (NH_3_ 75 μg/dL). However, it was noted that serum HBV DNA decreased. methylprednisolone (mPSL) pulse therapy of 1000 mg/day was added from day 24 to 26. The levels of liver enzymes gradually decreased and HBsAg became negative on day 31. On day 40, he was discharged and TAF was continued. Regular monitoring of the eGFR, urinary β2 microglobulin (U‐β2MG), and % tubular reabsorption of phosphate (%TRP) did not reveal renal dysfunction (Fig. 1b). Follow‐up monitoring showed normal levels of ALT and AST, and serum HBV DNA and HBsAg remained negative.

### Retrospective study

The characteristics of patients with HBV reactivation who received NA therapy are shown in Table 1. There was no significant difference between the ETV group (66 patients) and the TAF group (11 patients) with regard to age, sex, hepatitis B status, genotype, HBV DNA, ALT, HBeAg status, HBsAg titer, eGFR, original diseases, and purpose of treatment. The duration of treatment of the ETV group was significantly longer than that of the TAF group (ETV; 1120 (126–3278) days, TAF; 216 (128–567) days, *P* < 0.001).

**Table 1:** Baseline characteristics of patients who received entecavir (ETV) or tenofovir alafenamide (TAF) for hepatitis B virus (HBV) reactivation

	ETV (*n* = 66)	TAF (*n* = 11)	*P* value
Age (years): Median	68 (39–87)	69 (52–81)	0.855
Sex: Male/female	34/32	7/4	0.528
Hepatitis B status: Carrier/previous infection	50/16	7/4	0.462
Genotype: B/C/unknown	14/22/30	0/3/8	0.159
HBV DNA (logIU/mL): Median	3.1 (0–8.3)	2.3 (0–9.1)	0.498
ALT (U/mL): Median	18.5 (7–1363)	17.0 (11–1489)	0.393
HBeAg: Positive/negative/missing	9/44/13	2/8/1	0.787
HBs Ag (IU/mL): Median	280.00 (0.00–24 114.97)	398.66 (0.005–113 000)	0.999
eGFR (mL/min/1.73 m^2^): Median	72.9 (4.1–129.5)	70.9 (35.3–99.3)	0.856
Treatment duration (days): Median	1120 (126–3278)	216 (128–567)	<0.001
Original diseases: Malignant lymphoma/other cancer/others (rheumatoid arthritis, interstitial pneumonia, sudden sensorineural hearing loss, etc.)	16/29/21	3/5/3	0.999
Purpose of treatment: Prevention/reactivation (de novo hepatitis)	50/16 (4)	8/3 (2)	0.999

ALT, alanine aminotransferase; eGFR, estimated glomerular filtration rate.

**Figure 2:**
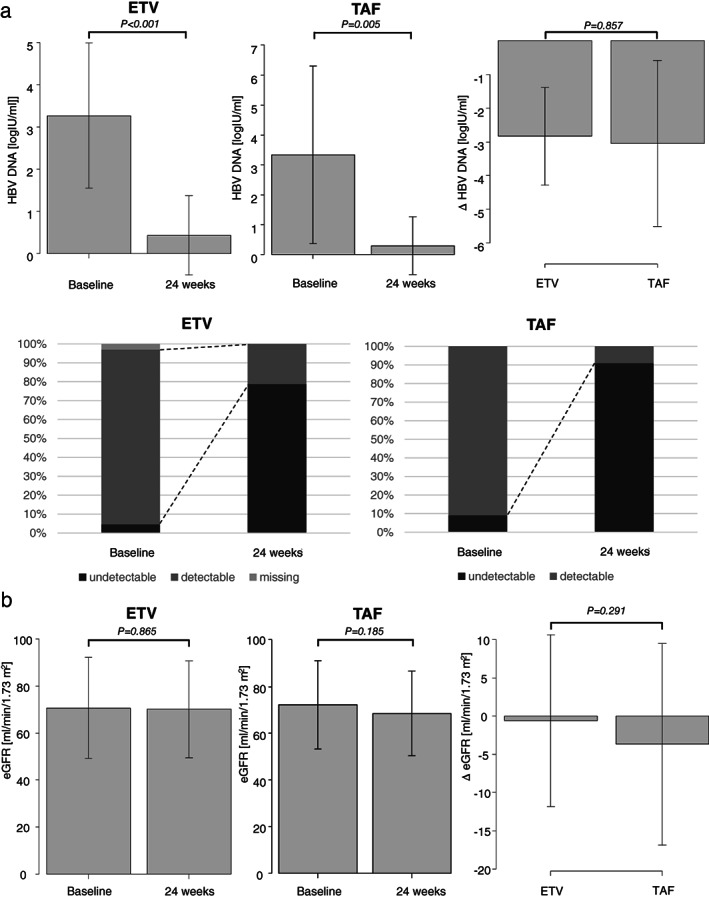
Antiviral effect and safety of entecavir (ETV) and tenofovir alafenamide (TAF) against HBV reactivation. (a) Mean serum hepatitis B virus (HBV) DNA levels of both groups before treatment (at week 0) and at week 24. The decrease in HBV DNA levels (delta HBV DNA; Δ HBV DNA) was compared. The proportion of patients who achieved undetectable HBV DNA at weeks 0 and 24. ETV: (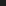), undetectable; (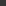), detectable; (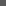), missing. TAF: (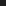), undetectable; (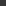), detectable. (b) Mean levels of estimated glomerular filtration rate (eGFR) of both groups before treatment (at week 0) and at week 24. The decrease in the eGFR levels (delta eGFR; Δ eGFR) was compared.

First, we investigated the antiviral efficacy of ETV and TAF for patients with HBV reactivation. Serum HBV DNA significantly decreased from week 0 to week 24 in patients treated with ETV (3.27 ± 1.72/0.43 ± 0.94 Log IU/mL, at week 0/24, respectively, *P* < 0.001) and TAF (3.34 ± 2.98/0.29 ± 0.96 Log IU/mL at week 0/24, respectively, *P* = 0.005). There was no significant difference in the decrease of serum HBV DNA between the ETV and TAF groups (−2.83 ± 1.45 *vs* −3.04 ± 2.47; *P* = 0.857) (Fig. 2a). At week 24, HBV DNA was undetectable in the serum of the patients in both groups (ETV: 78.8 *vs* TAF: 90.9%; *P* = 0.681).

Next, we investigated the safety of both drugs. There was no significant decrease in the eGFR of patients who received ETV (70.7 ± 21.6/70.1 ± 20.7 mL/min/1.73 m^2^, at week 0/24, respectively, *P* = 0.865) or TAF (72.2 ± 19.0/68.6 ± 18.2 mL/min/1.73 m^2^, at week 0/24, respectively, *P* = 0.185) (Fig. 2b). There was no significant difference in the decrease in eGFR between the ETV and TAF groups (−0.62 ± 11.22 *vs* −3.67 ± 13.19; *P* = 0.291) (Fig. 2b). Based on these results, both drugs are safe and effective.

Among the 11 patients who received TAF for HBV reactivation, two patients were treated for de novo hepatitis, one patient was treated for HBV reactivation with serum HBV DNA elevation, and eight patients took TAF for prophylaxis against HBV reactivation (Table 2). The two patients with de novo hepatitis suffered from malignant lymphoma and were treated with the R‐CHOP regimen. No recurrence of de novo hepatitis was observed. All the other patients also successfully suppressed HBV reactivation with TAF.

**Table 2:** Clinical background and course of patients who received tenofovir alafenamide (TAF) for hepatitis B virus (HBV) reactivation

Patient number	Age	Sex	Disease	Chemotherapy or immune suppression therapy	HBV status at baseline	Purpose of TAF treatment	Outcome
1	78	Male	Malignant lymphoma	Rituximab Cyclophosphamide Doxorubicin Vincristine Prednisolone	Previous infection	Reactivation with ALT flare	No recurrence. Chemotherapy‐free. TAF treatment in progress.
2	66	Male	Malignant lymphoma	Rituximab Cyclophosphamide Doxorubicin Vincristine Prednisolone	Previous infection	Reactivation with ALT flare	No recurrence. Ongoing chemotherapy. TAF treatment in progress with small amount of PSL.
3	81	Female	Microscopic polyangitis	Prednisolone Methotrexate Tacrolimus	Previous infection	Reactivation	No recurrence. Ongoing chemotherapy. TAF treatment in progress.
4	71	Male	Malignant lymphoma	Rituximab Cyclophosphamide Doxorubicin Vincristine Prednisolone	Previous infection	Prophylaxis	No reactivation after temporary HBV DNA elevation due to TAF discontinuation. Chemotherapy‐free. TAF treatment in progress after 2 months of cessation.
5	64	Male	Chronic lymphocytic leukemia	Rituximab Cyclophosphamide Doxorubicin Vincristine Prednisolone	Carrier	Prophylaxis	No reactivation. Chemotherapy‐free. TAF treatment in progress.
6	69	Male	Chronic lymphocytic leukemia	Rituximab Pirarubicin Cyclophosphamide Vincristine Prednisolone	Carrier	Prophylaxis	No reactivation. Ongoing chemotherapy. TAF treatment in progress.
7	76	Male	Cholangiocellular carcinoma	Gemcitabine Cisplatin	Carrier	Prophylaxis	No reactivation. Ongoing chemotherapy. TAF treatment in progress.
8	72	Female	Lung cancer	Tegafur‐uracil	Carrier	Prophylaxis	No reactivation. Ongoing chemotherapy. TAF treatment in progress.
9	52	Female	Breast cancer	Adriamycin Cyclophosphamide	Carrier	Prophylaxis	No reactivation. Ongoing chemotherapy. TAF treatment in progress.
10	59	Female	Rheumatoid arthritis	Methotrexate Prednisolone	Carrier	Prophylaxis	No reactivation. Ongoing chemotherapy. TAF treatment in progress.
11	53	Male	Sudden sensorineural hearing loss	Prednisolone	Carrier	Prophylaxis	No reactivation. Chemotherapy‐free. TAF treatment in progress.

ALT, alanine aminotransferase; PSL, prednisolone.

## Discussion

This study provides evidence of the efficacy and safety of TAF for HBV reactivation and de novo hepatitis based on real‐world data. Although TAF has been approved for use for hepatitis B, there are no studies evaluating its efficacy as a prophylactic and antiviral agent against HBV reactivation. As a countermeasure against HBV reactivation, monitoring should be done for all patients who have reactivation risks with the intent of initiating on‐demand antiviral therapy at the first sign of reactivation. However, diagnosing de novo hepatitis early during reactivation is sometimes difficult due to the time‐lag of serum HBV DNA. In our presented case, administration of TAF proved useful in preventing HBV replication. The steroids also played a supplementary role in suppressing severe inflammation and hepatitis B flare, as previous reports have stated.^24^, ^25^ Treatment using these drugs can avoid a fatal outcome in high‐risk patients (Fig. 1).

TAF is a novel NA whose absorption is not affected by food, unlike ETV, which should be taken between meals. This difference has improved drug adherence and satisfaction of patients who were previously being given ETV.^26^ This is important especially for patients with HBV reactivation risks who must undergo cytotoxic or immunosuppressive therapy. In HBV prophylaxis, adherence to treatment is as important as the drug's safety and efficacy.

In this study, the baseline characteristics were similar between the ETV and TAF treatment groups (Table 1). The treatment duration was longer in the ETV group because the approval date of the drug was earlier. We also investigated serum HBV DNA levels and eGFR decline as side effects. There was no significant difference in the decrease of serum HBV DNA and in the proportion of patients who achieved undetectable HBV DNA between the two groups (Fig. 2a). Furthermore, both drugs were well tolerated. The mean change in the eGFR from baseline to that at 24 weeks was similar for both groups (Fig. 2b). These data indicated that TAF was effective in terms of suppressing HBV DNA replication while maintaining eGFR.

There are several limitations in this study. This study showed the efficacy and safety of TAF against HBV reactivation. However, the observation period may have been insufficient to describe the long‐term safety and effects. Furthermore, the course after discontinuing NA has not been described. A longer observation period and a larger number of participants are needed. Another limitation is that we could not investigate the cost‐effectiveness of the drugs. There are only a few studies regarding cost‐effectiveness,^27^ and this factor has to be considered in the future.

In conclusion, TAF and ETV are safe and effective against HBV reactivation. TAF may lead to better outcomes due to its accessibility and better patient adherence.
